# Enhancing Functionality of Epoxy–TiO_2_-Embedded High-Strength Lightweight Aggregates

**DOI:** 10.3390/polym12102384

**Published:** 2020-10-16

**Authors:** Taekyung Lim, Jeong Hui Lee, Ju-Hyun Mun, Keun-Hyeok Yang, Sanghyun Ju, Sang-Mi Jeong

**Affiliations:** 1 Department of Physics, Kyonggi University, Suwon, Gyeonggi-do 16227, Korea; tklim@kgu.ac.kr (T.L.); jhwanna@naver.com (J.H.L.); 2 Department of Architectural Engineering Graduate School, Kyonggi University, Suwon, Gyeonggi-do 16227, Korea; mjh@kgu.ac.kr (J.-H.M.); yangkh@kgu.ac.kr (K.-H.Y.)

**Keywords:** epoxy–titanium oxide-embedded, high-strength lightweight aggregates, NO_x_ and SO_x_ reduction

## Abstract

With the increasing trend of high-rise, large-scale, and functional modern architectural structures, lightweight aggregate (LWA) concrete that exhibits excellent strength and high functionality has garnered active research attention. In particular, as the properties of concrete vary considerably with the raw materials and the proportions of aggregates in the mix, in-depth research on weight reduction, strength improvement, and functional enhancements of aggregates is crucial. This study used the negative pressure coating of a mixed solution comprising epoxy (mixture of epoxy resin and crosslinker), hyper-crosslinked polymer, and titanium oxide (TiO_2_) nanoparticles on the LWA, and achieved an improvement in the strength of the LWA as well as a reduction in air pollutants such as NO_x_ and SO_x_. Compared to a normal LWA with an aggregate impact value (AIV) of 38.7%, the AIV of the proposed epoxy–TiO_2_-embedded high-strength functional LWA was reduced by approximately half to 21.1%. In addition, the reduction rates of NO_x_ and SO_x_ gases resulting from the photocatalytic properties of TiO_2_ nanoparticles coated with epoxy were approximately 90.9% and 92.8%, respectively. Epoxy–TiO_2_, embedded in LWAs through a mixture, exhibited stability, high strength, and a reduction in air pollutant characteristics, despite repeated water washing. The LWA proposed herein offers excellent structural and functional properties and is expected to be used in functional lightweight concrete that can be practically applied in high-rise and large-scale architectural structures.

## 1. Introduction

Recently, lightweight aggregate (LWA) concrete, which is lightweight yet strong, has received increasing attention owing to the accelerating trend of high-rise, large-scale, non-standardized, and specialized building structures. When LWAs are mixed with cement, the aggregates act as reinforcements and fillers for concrete, improving its strength and reducing its weight. Therefore, the self-weight of the structure and the cross section of the structural members can be reduced along with improvements in the exclusive space of the structure and earthquake resistance.

In general, LWAs are divided into natural LWAs using natural minerals and artificial LWAs made of fly ash, cinder ash, and bottom ash. Recently, artificial LWAs have received attention from precast concrete industries, such as panels with higher insulation and structural elements including walls and slabs, because the lower density of LWA concrete can lead to lower-cost transportation and easier handling in the construction site. However, compared to natural LWAs, artificial LWAs generally have a low strength due to high moisture absorption [1]. The specific gravity, strength, and durability of LWAs varies depending on the particle shape, surface structure, porosity distribution, raw materials, and manufacturing process. Therefore, when a penetration fracture occurs within the artificial LWA concrete, the resistance effect of the LWAs lowers resulting in a brittle fracture. Accordingly, various studies have been conducted to develop artificial LWAs with enhanced strength and durability. The density and porosity of LWAs were controlled in the process of sintering LWAs at high temperature by changing the content of materials, such as foamed blast furnace slag, expanded vermiculite, and pelletized slag, that can sufficiently generate gas [2,3]. Binder materials, such as bentonite, metakaolin, and fly ash, with pyroplasticity properties at high temperature are mixed to strengthen the bonding strength of LWA materials [4,5,6,7]. In addition, in order to increase the strength of the LWAs, physical or chemical methods were performed on the LWA: physical methods include mechanical grinding/churning, physical heating, and heat grinding [8] and chemical methods include presoaking of LWA in various acidic/basic solutions, biodeposition, chemical grouting, carbonation, polymer treatment, and nano-modification of the aggregate surface [8,9,10].

It is advantageous that LWAs with various functionalities can be produced simply by introducing a few additives to the raw material mixture. For instance, TiO_2_, a representative photocatalytic material, was applied to LWAs to impart NO_x_ and SO_x_ reduction functionality [11,12,13,14,15]. It is well known that TiO_2_ is superior to other photocatalytic materials, such as ZnO, SnO_2_, Fe_2_O_3_, CuO, NiO, and CeO_2_, in its ability to remove NO_x_ and SO_x_ by the photo-decomposition principle under UV light irradiation [16,17]. In addition, the enhanced photodegradation performance of a cement-based composite by controlling the content of TiO_2_ in the cement-based composite [18], the impregnation and coating of sodium silicate on LWAs to provide self-healing properties [19], the improved skid-resistance of LWAs by controlling aggregate morphology through micro-surfacing [20], the hydrophobicity control of LWAs modified with sewage sludge [21], and the embedment of phase change materials into LWAs to improve thermal energy storage characteristics and reduce damage mitigation [9,22] have been studied. Although various studies are being conducted to develop artificial LWAs that can increase the strength and durability of aggregates and exhibit various functions, there has been minimal progress in the development of high-quality multi-functional artificial LWAs that can be applied to high-rise and large-scale buildings.

The aim of this study is to maintain the light weight of the aggregates while simultaneously enhancing the strength of the pores by bonding a hyper-crosslinked polymer (HCP) to the inner pores of LWAs. Accordingly, a solution containing epoxy and HCP was embedded in the inner pores of the LWA under negative pressure conditions, and crosslinking and polymerization were employed as coating methods for the post-treatment of the LWA. In addition, the reduction of NO_x_ and SO_x_ was also carried out by adding TiO_2_ nanoparticles (NPs) with photocatalytic properties, along with epoxy. The strength and NO_x_/SO_x_ reduction ability of the aggregates were confirmed based on the maintenance of the epoxy–TiO_2_ coating of the pores that were present in the inner and outer surfaces of the LWA which remained unaffected despite harsh external environment conditions such as rain. This study aims to introduce a new method to fabricate LWA that is structurally and functionally superior by addressing the brittle fracture problem, a fundamental problem of artificial LWAs, and by enhancing the pollutant-reduction functionality by targeting additional air pollutants.

## 2. Materials and Methods

### 2.1. Fabrication of Epoxy–TiO_2_-Embedded High-Strength Functional LWA

High-strength functional LWA was fabricated by simultaneously embedding epoxy (mixture of epoxy resin and crosslinker; CM–ER–Thin, R&B Co. Ltd., Daejoen, South Korea) into the internal pores (~0.3–300 μm) of pristine artificial LWA (size ~13–15 mm, porosity~ 44%, NDlite-LWA, Korea South-East Power Co., Ltd., Jinju-si, South Korea) and by coating the external surface. The density and water absorption of LWA were 1.4 g/cm^3^ and 14.1%, respectively. To remove pollutants including dust on the surface of the artificial LWA, the pristine LWA was washed with running water and dried in air for 15 h. An epoxy solution was prepared by mixing epoxy in isopropyl alcohol (IPA, DAEJUNG, Siheung-si, South Korea) in six different ratios from 0 to 15 wt.%, and the crosslinker was fixed at 25 wt.% of epoxy resin. The ATR–FTIR spectrum of the mixture of epoxy resin and crosslinker showed the typical epoxy-related peaks, a stretching O–H peak of 3370.53 cm^−1^, a C–H stretching vibration peak of 2928.98 cm^−1^ of CH_3_, aromatic peaks of 1609.54 and 1506.62 cm^−1^, and C–O–C stretching peaks of 1229.58 and 1031.47 cm^−1^. Thereafter, the epoxy solution and TiO_2_ NPs (size ~300 nm, density ~4.26 g/mL (at 25 °C), rutile structure, ReagentPlus^®^, Sigma–Aldrich, St. Louis, MI, USA) were mixed at a mass ratio of 10:1 and were sonicated to prepare an epoxy–TiO_2_ composite solution. The prepared epoxy–TiO_2_ composite solution was embedded in the LWA under normal or negative pressure coating conditions. In the case of the normal pressure coating, LWAs were dipped in an epoxy–TiO_2_ composite solution for 60 min at 20 °C under normal pressure conditions (1 atm). Subsequently, the LWAs were removed from the solution and dried in air for 8 h. For the negative pressure coating, the epoxy–TiO_2_ composite solution containing LWAs was placed in a vacuum oven (SH–VDO–08NG, SH SCIENTIFIC, Sejong, South Korea) and held at a negative pressure of 600 Torr for 30 min. Then, the LWAs were removed from the solution and dried for 8 h under room temperature (20 °C) and normal pressure conditions (1 atm).

### 2.2. Material Properties of Aggregates

To determine the amount of epoxy–TiO_2_ coated on the pristine LWA, the LWA was weighed and its aggregate impact value (AIV) was measured via an aggregate impact testing machine (HJ–2230, Heungjin Testing Machine Co. Ltd., Gimpo-si, South Korea) before and after the epoxy–TiO_2_ coating. According to BS 812–112 (Testing aggregates-Part 112: Methods for determination of AIV), the method of the determination of AIV is obtained by vertically dropping a weight of 14 kg from 381 mm above the surface of the aggregates 15 times to crush them. Thereafter, the crushed aggregates are sieved using a sieve with a 2.36 mm-sized aperture, and the weight difference of the aggregates before and after the crushing is calculated; the value is converted into a percentage. Attenuated total reflection Fourier-transform infrared (ATR-FTIR) spectroscopy (Nicolet iS50, Thermo-Fisher Scientific, Waltham, MA, USA) was employed to determine if the epoxy was evenly embedded into the LWA pores. Field emission scanning electron microscopy (FE–SEM; JSM–7610F PLUS, JEOL, Tokyo, Japan) and energy-dispersive X-ray spectrometry (EDS; Inca x–sight model 7557, Oxford Instruments, Abingdon, UK) were employed to determine the shape of the inner side of the LWA and the presence of coated TiO_2_.

### 2.3. SO_x_ and NO_x_ Adsorption Properties of Aggregates

A NO_x_/SO_x_ gas analysis system (Invisible Co., Suwon, South Korea) was used to determine the effect of reducing NO_x_ and SO_x_ gases on the epoxy–TiO_2_-embedded LWA. A total of 500 g of epoxy–TiO_2_-embedded LWA was placed in an open box with the dimensions 705 mm (width, W) × 210 mm (length, L) × 105 mm (height, H); thereafter, it was placed inside the main chamber of the NO_x_/SO_x_ gas analysis system. Subsequently, UV light (UV intensity ~1.2 mW/cm^2^) was irradiated at a distance of 220 mm from the LWA. The amount of NO and SO_2_ gas injected into the chamber was controlled using a mass flow controller (3660, KOFLOC, Tokyo, Japan) and readouts (MR-300, MJ technics, Incheon, South Korea). For the measurement of NO_x_ reduction, 303.0 μmol/mol of NO and N_2_ gases was injected into the chamber at a rate of 3.8 sccm and 3 slm, respectively. For the measurement of SO_x_ reduction, 300.0 μmol/mol of SO_2_ and N_2_ gases was injected into the chamber at a rate of 19.5 sccm and 0.5 slm, respectively. After 2 h of injection, the NO or SO_2_ inside the chamber attained a concentration of 1 ppm, which was maintained for another 3 h. Then, the NO_x_ or SO_x_ concentration inside the chamber was measured under UV light irradiation. A NO_x_ analyzer (MEZUS–210, Kemik Corporation, Seongnam-si, South Korea) and a SO_x_ analyzer (MEZUS–110, Kemik Corporation, Seongnam-si, South Korea) were used for the measurement of concentrations.

### 2.4. Changes in NO_x_ Removal Efficiency of Epoxy–TiO_2_ Embedded LWA According to Water Washing

After washing the epoxy–TiO_2_-embedded LWA, the following process was conducted to confirm the change in NO_x_ removal efficiency. First, 500 g of epoxy–TiO_2_-coated LWA that was tested for NO_x_ removal was transferred to a sieve with an aperture of 2 mm (diameter ~175 mm). After placing a spray gun (AS ONE) at a height of 300 mm above the epoxy–TiO_2_-embedded LWA, water was sprayed on the entire anterior surface of the epoxy–TiO_2_-embedded LWA at a rate of 1250 mL/min for 10 min. The wet epoxy–TiO_2_-embedded LWA was thoroughly air-dried at room temperature for 12 h. Finally, the NO_x_ removal effect of the dried epoxy–TiO_2_-coated LWA was measured.

## 3. Results and Discussion

HCP is highly applicable in energy and environment sectors such as for gas storage, carbon capture/removal, pollutant isolation/removal, and catalysts due to the advantages of a variety of synthesis methods, easy functionalization, high surface area, and mild reaction conditions [23,24,25,26]. When HCP bonds to the inner pores of LWA, the strength of the LWA is enhanced by the physical/chemical bonding between the HCP and the porous surface and the light weight of the LWA is maintained. A method for improving the functionality of LWA is proposed here where it adsorbs and decomposes the air pollutants SO_x_ and NO_x_ by uniformly mixing HCP with TiO_2_.

**Figure 1:**
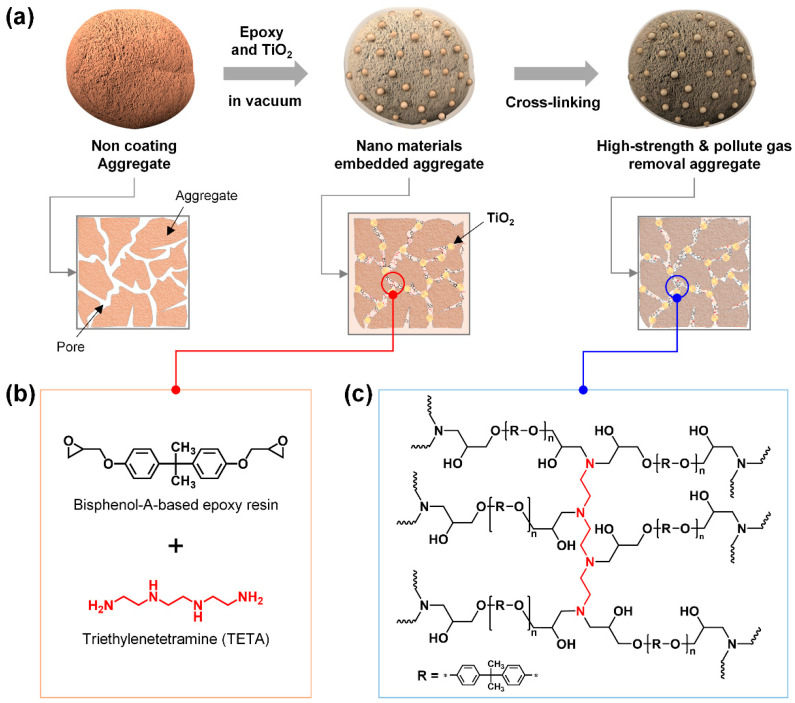
Fabrication of an epoxy–TiO_2_-embedded lightweight aggregate (LWA). (**a**) Schematic diagram of embedding hyper-crosslinked epoxy into the LWA. (**b**) Chemical structure of the epoxy resin and the crosslinker being filled into the pores of the LWA. (**c**) Chemical structure of the post-crosslinked epoxy polymer.

Figure 1a shows how HCP can be embedded into LWA by introducing monomers and crosslinkers that form a microporous network with the inner pores of LWA under negative pressure conditions through crosslinking and polymerization post-treatments. As HCP is a permanent microporous polymer material, it can form a polymer chain network via the chemical reaction of the monomers and crosslinkers. Generally, polymers occupy a large volume relative to the molecular weight and due to the entanglement of long polymer chains, penetration into small pores is difficult. Therefore, it is necessary to control the material mixing and reaction time to ensure the HCP can penetrate the small pores of monomer and oligomer states, so that crosslinking and polymerization reactions can continue. In this study, epoxy resin, a representative HCP, was used to facilitate crosslinking and polymerization under mild reaction conditions. Here, resin, a high-viscosity material that can be converted to polymers through crosslinking or polymerization, and a crosslinker (or hardener) are mixed and hardened for use. Since the epoxide groups are chemically bonded to the pores of the inner surface of the LWA during the crosslinking reaction and polymerization, the strength of the LWA is improved through the additional physical/chemical bonding between epoxy and the pores, while the light weight of the LWA is maintained. In addition, TiO_2_ NPs as a photocatalyst were mixed with an epoxy solution to remove NO_x_ and SO_x_, which are typical air pollutants. During the preparation of the epoxy–TiO_2_ composite solution, IPA was added to the high-viscosity epoxy resin to lower the viscosity and reduce the reaction rate with the crosslinker; thereafter, the TiO_2_ NPs were mixed and ultrasonic homogenization was performed. When the prepared epoxy–TiO_2_ composite solution is embedded in the LWA, the composite solution cannot penetrate into the pores owing to the presence of air, and therefore it only coats the outer surface of the aggregates. In order to tackle this problem, a vacuum state (600 Torr) is maintained while dipping the LWA in the composite solution; this causes the air inside the LWA to escape and the composite solution can therefore easily penetrate into the pores of the LWA through a capillary phenomenon.

Figure 1b shows the chemical structures of a bisphenol-A-based epoxy resin with epoxide functional groups, an HCP and triethylenetetramine (TETA) with four amine functional groups, and a crosslinker. Epoxy resin is a small molecule or oligomer with a lower molecular weight and volume compared to crosslinked polymers; therefore, it can easily penetrate into the pores of LWA. An epoxy resin and a crosslinker are introduced into the LWA pores, resulting in polymerization by a crosslinking reaction, and the formation of a network of polymer chains with micropores inside the LWA pores. (Figure 1c). Simultaneously, the epoxide functional groups of epoxy resin and the amine functional groups of TETA chemically react with the ceramic components (SiO_2_, Al_2_O_3_, Fe_2_O_3_, CaO, MaO, and Na_2_O) in the LWA, and due to bonding with the porous surface of the aggregates, the strength of the LWA is enhanced. Despite the addition of TiO_2_ NPs with photocatalytic properties into the epoxy, the inflow of SO_x_ and NO_x_ gases remains unaffected due to the microporous network structure, an inherent property of HCP, and therefore the functionality of SO_x_ and NO_x_ gas decomposition is imparted [27,28,29,30].

**Figure 2:**
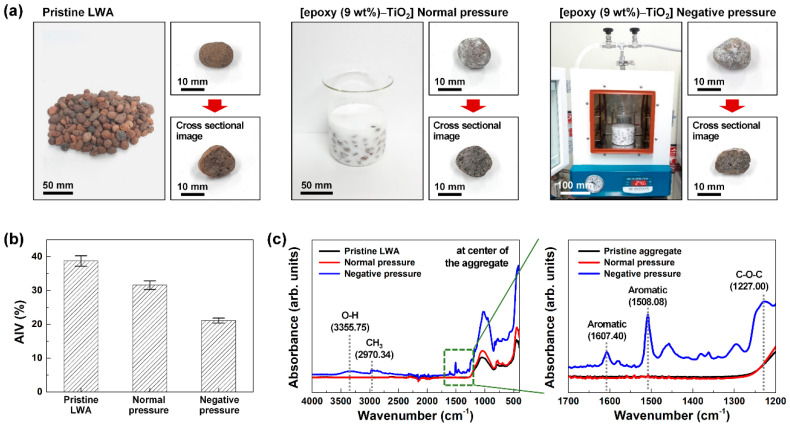
Comparison of LWA properties according to coating conditions. (**a**) Surface and cross-section images, (**b**) aggregate impact value, (**c**) ATR–FTIR spectra of LWA cross-section of pristine LWA, LWA coated with epoxy–TiO_2_ under normal pressure, and LWA coated with epoxy–TiO_2_ under negative pressure.

Figure 2 displays the difference in strength and the degree of internal penetration of the LWA according to the method of coating the aggregates with epoxy–TiO_2_ solution while fabricating a high-strength LWA for the adsorption of air pollutants. An aggregate impact testing machine which compares the difference between impact resistance and resistance due to static compressive load was used to measure the LWA strength. The hardness of the LWA can be determined by measuring the impact resistance and the amount of crushed LWA. In addition, the degree of penetration of epoxy–TiO_2_ into the LWA was obtained by crushing the coated LWA and examining the central part of the LWA through ATR–FTIR spectroscopy.

Figure 2a shows the pristine LWA, the LWA coated with epoxy–TiO_2_ under normal pressure, and the LWA coated with epoxy–TiO_2_ under negative pressure (600 Torr). The air present in the pores of the LWA hinders the penetration of the epoxy–TiO_2_ composite solution into the pores under normal pressure. Therefore, the coating of epoxy–TiO_2_ on the LWA was conducted under a negative pressure condition of 600 Torr in order to remove the air present in the LWA pores. As the brown-colored pristine LWA was coated with epoxy–TiO_2_, the overall appearance of white TiO_2_ NPs was displayed on a gray background. When examining the change in strength according to the coating conditions of the LWA, the AIV value of 38.7% ± 1.5% for the pristine LWA decreased to 31.6% ± 1.3% (normal pressure coating) and 21.1% ± 0.7% (negative pressure coating) according to the epoxy–TiO_2_ coating (Figure 2b). The results confirmed that the epoxy–TiO_2_ coating further strengthened the aggregates; in particular, the negative pressure coating reduced the AIV by ~45%, compared with the pristine LWA. This is because the epoxy coated on the porous surface of the LWA through crosslinking and polymerization acts as a binder. The epoxy coat on the porous surface of the LWA causes a firm bonding that can absorb any external impact on the aggregates, thus increasing their strength.

Figure 2c shows the ATR–FTIR spectra results of the central part of the LWA under the coated conditions. The pristine LWA and epoxy–TiO_2_-coated LWA under normal pressure conditions showed no epoxy-related peaks. On the contrary, in the LWA coated with epoxy–TiO_2_ under negative pressure conditions, a stretching O–H peak of 3355.75 cm^−1^ of epoxy, a C–H stretching vibration peak of 2970.34 cm^−1^ of CH_3_, aromatic peaks of 1607.40 and 1508.08 cm^−1^, and a C–O–C stretching peak of 1227.00 cm^−1^ were observed [31]. These results demonstrate that in the case of the normal pressure coating, epoxy is coated only on the surface of the LWA, and in the case of the negative pressure coating, epoxy effectively penetrates into the interior of the LWA and bonds tightly to the porous surface, thereby increasing its strength.

**Figure 3:**
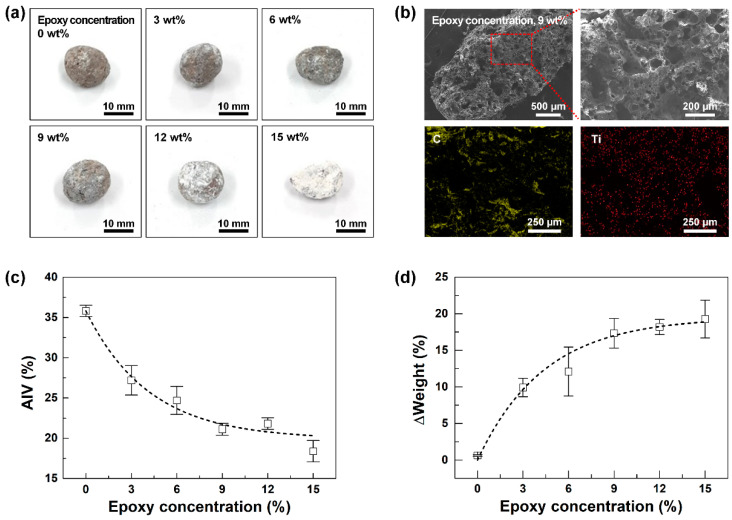
Optimization of LWA properties according to the concentration of epoxy–TiO_2_ composite solution. (**a**) Photo image of the epoxy–TiO_2_-embedded high-strength functional LWA according to six epoxy concentration conditions (0, 3, 6, 9, 12, and 15 wt.%). (**b**) FE–SEM image and EDS elemental image of C and Ti of 9 wt.% epoxy–TiO_2_-embedded high-strength functional LWA. (**c**) Aggregate impact value (AIV). (**d**) Weight changes of the epoxy–TiO_2_-embedded high-strength functional LWA according to the change in epoxy coating concentrations.

When the epoxy–TiO_2_ coating was applied to the LWA, a difference in the strength of the epoxy–TiO_2_-embedded high-strength functional LWA was observed depending on both the concentration of epoxy (epoxy resin and crosslinker) and the coating method. The concentration of the epoxy–TiO_2_ composite solution was changed by increasing the concentration of epoxy (epoxy resin and crosslinker) to 0, 3, 6, 9, 12, and 15 wt.% while keeping the amount of TiO_2_ NPs constant. Note that the ratio of epoxy resin to crosslinker was equal to 4:1. Figure 3 shows the properties of the functional LWA according to the concentration of epoxy in the epoxy–TiO_2_ composite solution coated on the LWA under negative pressure. TiO_2_ was mixed with an epoxy solution containing IPA and epoxy at a mass ratio of 10:1 to ensure a constant TiO_2_ ratio in the total epoxy–TiO_2_ composite solution. Figure 3a shows the photo image of the epoxy–TiO_2_-embedded high-strength functional LWA coated with a solution prepared using varying concentrations of epoxy at 0, 3, 6, 9, 12, and 15 wt.% with respect to the total epoxy–TiO_2_ composite solution. Epoxy not only acts as a binder in the inner pore surface of the aggregates, but also positions TiO_2_ ensuring it is stably embedded along the inner and outer surfaces of the aggregates, as shown by the uniformly distributed white TiO_2_ NPs occurring as the pristine LWA is coated with epoxy–TiO_2_. Furthermore, when the concentration of epoxy in the epoxy–TiO_2_ solution exceeds 12 wt.%, the viscosity increases as the amount of epoxy resin increases; the rate of crosslinking accelerates with the increase in the concentration, resulting in a drastic increase in the molecular weight of the crosslinked epoxy polymer. In this case, the volume of the epoxy polymer increases, making it difficult for the solution to penetrate the pores of the LWA; as the solubility of the epoxy polymer decreases, phase separation occurs and the layers are separated [32]. In addition, as the dispersion of epoxy and TiO_2_ in the epoxy–TiO_2_ solution decreased, epoxy–TiO_2_ was not uniformly coated on the inside and on the surface of the LWA but rather showed an agglomerated form.

An EDS measurement was conducted to investigate whether the epoxy–TiO_2_ was uniformly distributed inside the LWA. Figure 3b shows the results of the FE–SEM and EDS measurements of the inner side of the coated LWA after it was crushed under negative pressure with an epoxy–TiO_2_ solution containing 9 wt.% epoxy. FE–SEM images showed that the epoxy–TiO_2_ penetrated into the pores inside the LWA. Elemental C and Ti measured by EDS mapping showed that the epoxy–TiO_2_ was uniformly distributed inside the LWA.

Figure 3c,d show the trend in the changes of the AIV and the weight of the epoxy–TiO_2_-embedded high-strength functional LWA according to the epoxy concentration. AIV is an index to examine the strength of LWAs. To produce concrete with higher compressive strength and a longer lifespan, the use of LWAs with higher fracture resistance is essential. As the epoxy concentration increased, the AIV of the epoxy–TiO_2_-embedded high-strength functional LWA decreased from 35.8% ± 0.7, 27.2% ± 1.8%, 24.7% ± 1.7%, 21.1% ± 0.7%, and 21.8% ± 0.7% to 18.4% ± 1.3% (0, 3, 6, 9, 12, and 15 wt.%, respectively). (Figure 3c) As the epoxy concentration increased, the amount of epoxy–TiO_2_ that penetrated into the LWA also increased, and the strength of the aggregates increased owing to the binding and shock absorption properties of epoxy. In addition, as shown in the results of Figure 3d, as the epoxy concentration increased, the amount of epoxy–TiO_2_ in the epoxy–TiO_2_-embedded high-strength functional LWA increased, resulting in a gradual increase in weight from 0.6% ± 0.1%, 9.9% ± 1.3%, 12.1% ± 3.3%, 17.3% ± 2.0%, and 18.2% ± 1.0% to 19.3% ± 2.6% (0, 3, 6, 9, 12, and 15 wt.%, respectively). With an epoxy concentration of 9 wt.%, an optimal coating condition that does not cause agglomeration of epoxy–TiO_2_, the AIV decreased by approximately 50%, while only 0.2 times the weight was gained.

TiO_2_ NPs contained in the epoxy–TiO_2_-embedded high-strength functional LWA are typical photocatalytic materials and have the ability to decompose and reduce NO_x_ and SO_x_ gases when irradiated with UV light. TiO_2_, with a band gap of 3.2 eV, absorbs UV light at wavelengths below 386 nm, and excites electrons from the valence band (VB) to the conduction band (CB). Thus, holes (h^+^) are formed in the VB, whereas electrons (e^−^) are generated in the CB. The VB hole reacts with water molecules adsorbed on the aggregates to produce hydroxyl radicals (•OH). Meanwhile, electrons in CB reduce oxygen in the atmosphere to generate superoxide species (•O_2_^−^). Hydroxyl radicals and superoxide species chemically react to transform NO_x_ gas to nitrates and SO_x_ gas to sulfite and sulfate [33,34,35].

**Figure 4:**
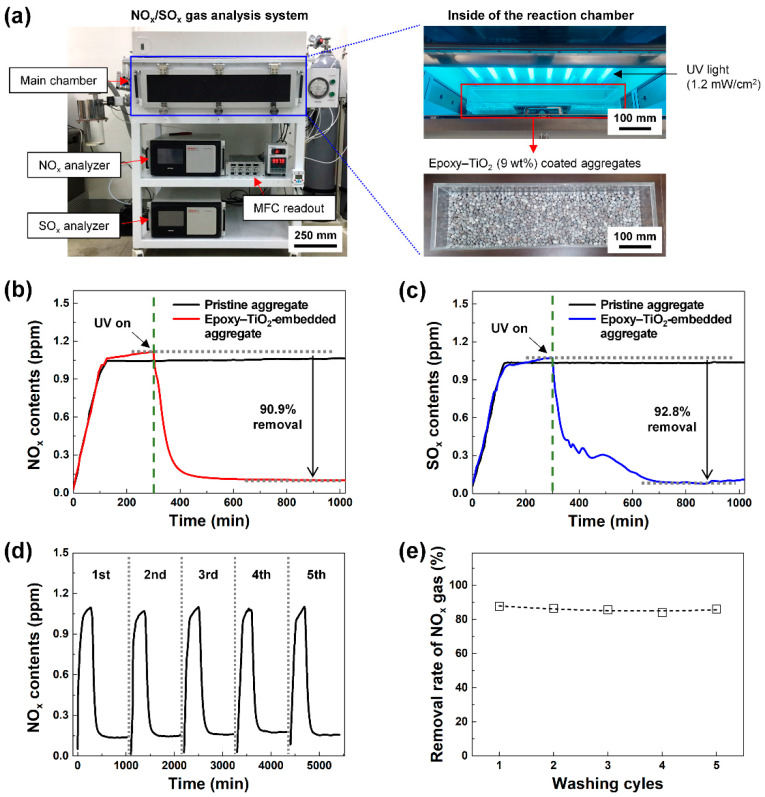
NO_x_/SOx adsorption capacities of the epoxy–TiO_2_-embedded high-strength functional LWA. (**a**) Images of the exterior and interior of the reaction chamber of the NO_x_/SO_x_ gas analysis system. (**b**) NO_x_ and (**c**) SO_x_ reduction properties of the pristine LWA and the epoxy–TiO_2_-embedded high-strength functional LWA. Dotted line is a baseline for extracting the maximum removal rate of NO_x_ and SO_x_ when UV is turned on. (**d**) The result of measuring the reduction of NO_x_ gas after 5 times of repeated water washing. (**e**) Changes in NO_x_ gas removal rate through the water washing cycles.

Figure 4a shows the NO_x_/SO_x_ gas analysis system. The epoxy–TiO_2_-embedded high-strength functional LWA was placed in the main chamber, and the NO_x_ or SO_x_ gas removal rate was measured under a UV intensity condition of 1.2 mW/cm^2^. The epoxy–TiO_2_-embedded high-strength functional LWA was fabricated with an epoxy concentration of 9 wt.% and showed excellent strength characteristics without causing the agglomeration of epoxy–TiO_2_. Figure 4b,c display the NO_x_ and SO_x_ reduction performance of the epoxy–TiO_2_–embedded high-strength functional LWA. After injecting NO_x_ or SO_x_ gas (1 ppm) into the NO_x_/SO_x_ gas analysis system, the concentration inside the chamber was kept constant for 5 h, and thereafter UV light was irradiated. Immediately, the concentration of NO_x_ or SO_x_ gas in the chamber began to decrease, and the maximum removal rates were 90.3% (NO_x_) and 92.8% (SO_x_). The concentration of NO_x_ gas in the chamber rapidly decreased until approximately 100 min after the UV irradiation was initiated, and then the concentration was kept constant at approximately 0.1 ppm for 12 h. The concentration of SO_x_ gas in the chamber continuously decreased until approximately 6 h after the UV irradiation was initiated, and then a concentration of approximately 0.1 ppm was maintained for the next 12 h.

As LWAs are applied to buildings exposed to the external environment, they are constantly exposed to harsh weather conditions such as precipitation. The fabricated epoxy–TiO_2_-embedded high-strength functional LWA exhibits stable durability under wet environments owing to the presence of epoxy in the composition of TiO_2_ NPs. Figure 4d,e show the continuity of the removal rate under repeated water washing. The NO_x_ gas reduction capacity of the epoxy–TiO_2_-embedded high-strength functional LWA was measured through five repeated washing cycles, and the NO_x_ gas removal rates observed were 87.7, 86.5, 85.7, 84.0, and 86.1%. Notably, the ~3% variation in this value is likely to be an error attributed to the degree of surface exposure of aggregates placed on the measuring equipment. Therefore, stable reduction characteristics were confirmed.

## 4. Conclusions

In summary, epoxy, an HCP, was used as a reactive binder for LWA surface bonding and as an external shock absorber in order to address the challenging drawback of the weak strength of LWAs. In addition, by adding TiO_2_ NPs to epoxy, a reduction in NO_x_ and SO_x_ (air pollutants) was noted. Although epoxy is coated on the surface of TiO_2_ NPs, NO_x_/SO_x_ and the gas decomposed by TiO_2_ NPs can be easily eliminated owing to the ability of HCP to form micropores during polymerization and crosslinking. Through the negative pressure coating method, epoxy–TiO_2_ completely penetrated the pores of the LWA, and by controlling the epoxy concentration from zero to 15 wt.%, an optimal condition was discovered at which the light weight of the LWA was maintained and its strength was enhanced. The AIV of the epoxy–TiO_2_-embedded high-strength functional LWA coated with 9 wt.% concentration, an optimal condition for no agglomeration of epoxy, is reduced by half to 21.1% when compared with the 38.7% of the pristine LWA. In addition, due to the photocatalytic properties of TiO_2_ NPs, it was confirmed that NO_x_ and SO_x_ gases were reduced by ~90.9% and ~92.8%, respectively, under UV light. Since TiO_2_ NPs are mixed with epoxy, stable NO_x_ gas reduction properties were maintained despite continuous and repeated washing. In the case of the negative pressure coating method used in this study, the surfaces of LWAs should be exposed to air. Accordingly, if the exposure of the surface of the LWAs is limited due to overlapping LWAs during the large-capacity coating process, it is difficult to remove air completely in the LWAs with a negative pressure of 600 Torr and some pores of the LWAs may not be coated. Therefore, in order to uniformly coat a large amount of LWAs, further research is needed on the development of equipment that can evenly expose a large amount of LWAs to the air in a negative pressure ambient. Epoxy–TiO_2_-embedded high-strength functional LWAs proposed in this study are expected to be widely applied in multifunctional LWA concrete as they offer excellent strength and durability as well as the functionality of reducing air pollution.
